# Characterization of an α 4/7-Conotoxin LvIF from *Conus lividus* That Selectively Blocks α3β2 Nicotinic Acetylcholine Receptor

**DOI:** 10.3390/md19070398

**Published:** 2021-07-17

**Authors:** Man Guo, Jinpeng Yu, Xiaopeng Zhu, Dongting Zhangsun, Sulan Luo

**Affiliations:** 1 Key Laboratory of Tropical Biological Resources of Ministry of Education, Key Laboratory for Marine Drugs of Haikou, School of Life and Pharmaceutical Sciences, Hainan University, Haikou 570228, China; guoman0927@hainanu.edu.cn; 2 Medical School, Guangxi University, Nanning 530004, China; jinpengyu2019@126.com (J.Y.); biozxp@163.com (X.Z.)

**Keywords:** α-CTx LvIF, rα3β2 nAChR, two-electrode voltage clamp, two-step oxidation

## Abstract

Nicotinic acetylcholine receptor (nAChR), a member of pentameric ligand-gated ion channel transmembrane protein composed of five subunits, is widely distributed in the central and peripheral nervous system. The nAChRs are associated with various neurological diseases, including schizophrenia, Alzheimer’s disease, Parkinson’s disease, epilepsy and neuralgia. Receptors containing the α3 subunit are associated with analgesia, generating our interest in their role in pharmacological studies. In this study, α-conotoxin (α-CTx) LvIF was identified as a 16 amino acid peptide using a genomic DNA clone of *Conus lividus (C. lividus)*. The mature LvIF with natural structure was synthesized by a two-step oxidation method. The blocking potency of α-CTx lvIF on nAChR was detected by a two-electrode voltage clamp. Our results showed that α-CTx LvIF was highly potent against rα3β2 and rα6/α3β2β3 nAChR subtypes, The half-maximal inhibitory concentration (IC_50_) values of α-CTx LvIF against rα3β2 and rα6/α3β2β3 nAChRs expressed in *Xenopus* oocytes were 8.9 nM and 14.4 nM, respectively. Furthermore, α-CTx LvIF exhibited no obvious inhibition on other nAChR subtypes. Meanwhile, we also conducted a competitive binding experiment between α-CTxs MII and LvIF, which showed that α-CTxs LvIF and MII bind with rα3β2 nAChR at the partial overlapping domain. These results indicate that the α-CTx LvIF has high potential as a new candidate tool for the studying of rα3β2 nAChR related neurophysiology and pharmacology.

## 1. Introduction

The nAChR, an important part of a cholinergic transmission of the central and peripheral nervous system and skeletal muscle neuromuscular junctions, is a classic nicotine-sensitive ligand-gated ion channel of the cysteine-loop superfamily, which is endogenously activated by acetylcholine [1,2]. According to the distribution of nAChR in different tissues, it can be divided into muscular and neural nAChRs [3]. The neural nAChR is one of the most concerned ion channels and membrane proteins. So far, researchers have cloned 14 different nAChR subunits have been identified in vertebrates, including α1–α10, β1–β4 [4,5], which can co-assemble to form multiple functional heteropentamers or homopentamers. The α3β2 nAChR is one of the important subtypes which is mainly distributed in the dorsal root ganglion and spinal cord [6]. Recent research shows that α3β2 nAChR is mainly related to pain and perception. The α3β2 nAChR located in the dorsal horn of the lumbar spinal cord and inhibited the release of glutamate from primary afferent C-fibers, subsequently reduced the sensitivity of rats to mechanical pain. Intrathecal injection of α3β2 nAChR antagonist decreased the pain threshold and enhanced the transmission of pain mechanical stimulation, however, accurate distribution and function of α3β2 nAChR still remains unknown [7].

The α-conotoxins (α-CTxs) are a unique group of neuropeptide toxins with small molecular weights and rich in disulfide bonds extracted from the venom of cone snails. They are generally composed of 11–20 amino acid residues. There are many kinds of α-CTxs, with various activities and complex structural changes. At present, α-CTxs are mainly classified by their highly conserved signal peptide sequence, combined with their pharmacological activities and cysteine patterns. The cysteine frame of α-CTx is CC-C-C, in which the two-loop rings form two pairs of disulfide bonds, Cys I-Cys III and Cys II-Cys IV. According to the different amount of amino acids between Cys II-Cys III and Cys III-Cys IV, α-CTxs can be divided into many subfamilies, such as α 3/5, α 4/7, α 4/6, α 4/4 and α 4/3 [8,9,10]. The α-CTxs would be competitive antagonists of nAChRs and an effective pharmacological tool to study the mechanism of ligand-receptor interactions. At the same time, the relationship between α-CTxs and various subtypes of nAChR has been studied extensively, which provides more understanding of the relationship between nAChR and some neurological diseases.

Previously, we found α 4/7-CTx LvIA with blocking rα3β2 and rα3β4 nAChRs and α4/4-CTx LvIB with blocking rα7 and rα6/α3β2β3 nAChRs from *C. lividus* in our laboratory, but the α-CTx LvIA had poor selectivity, and the α-CTx LvIB had poorly binding efficacy to the rα3β2 nAChR [11,12]. In this study, a new α 4/7-CTxs, named α-CTx LvIF, was identified from *C. lividus* in Hainan Province. It was found that α-CTx LvIF had a strong blocking effect on rα3β2 nAChR. Subsequently, we further detected its blocking effect and selectivity by a two-electrode voltage clamp. It provides an effective pharmacological tool to study the structure-activity relationship of rα3β2 nAChR, and rα3β2 nAChR-related diseases.

## 2. Results

### 2.1. DNA Cloning and Sequence Analysis of the α-CTx LvIF Precursor

**Figure 1:**
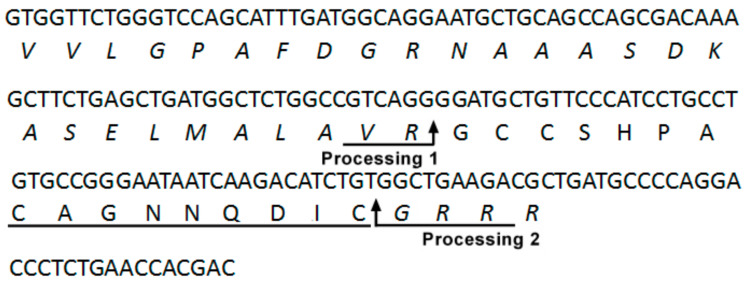
α-CTx LvIF pre-propeptide and encoded toxin. A putative proteolytic processing site 1 (R) and amidation processing site 2 (C) are indicated by the arrows, and the Pro-region is in italics. The first glycine following the C-terminal cysteine in the mature toxin is presumed to be processed to a C-terminal amide in α-CTx LvIF. The deduced mature toxin sequence of LvIF (the underlined region) is GCCSHPACAGNNQDIC# (#, C-terminal carboxamide).

A new gene was identified by exploiting the conserved feature of the α-CTx gene structure to yield a α 4/7-CTx. The mature peptide of α 4/7-CTx LvIF was truncated to a length of 16 amino acids and cleaved from the larger precursor protein by proteolytic cleavage and post-translational modifications. A 170 base pair sequence encoding for a 16 amino acid mature region with the sequence GCCSHPACAGNNQDIC#(# = represents C-terminal carboxamide) was identified. The precursor gene of α-CTx LvIF is shown in Figure 1. α-CTx LvIF displays a cysteine framework of type I (CC-C-C) and belongs to the gene Superfamily ‘A’. Other CTxs that display similar characteristics typically inhibit nAChRs, suggesting that α-CTx LvIF has similar pharmacological properties. Structurally, there are two conserved amino acids in the first loop (Ser and Pro), while the sequence of the second loop of α-CTx LvIF is divergent from other known members of the α-CTx family.

### 2.2. Oxidative Folding and Identification of α-CTx LvIF

**Figure 2:**
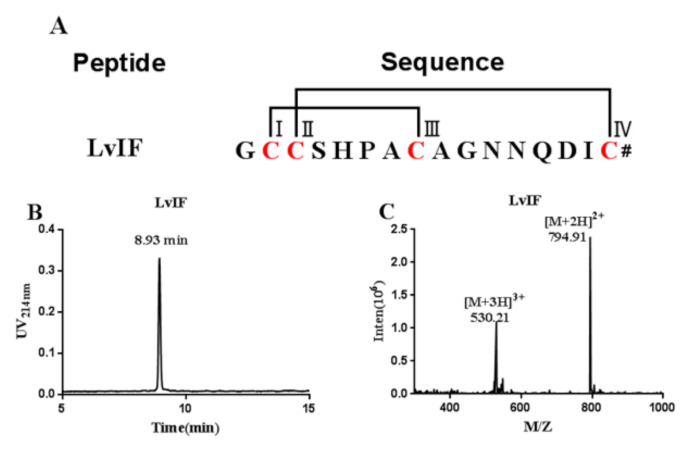
The Sequences, HPLC chromatograms and ESI-MS of α-CTx LvIF. (**A**) The cysteines are marked in red. # indicates a C-terminal carboxamide. (**B**) HPLC chromatogram of α-CTx LvIF. (**C**) ESI-MS data for α-CTx LvIF with an observed monoisotopic mass of 1589.8 Da.

The linear peptide corresponding to the deduced mature LvIF sequence was synthesized via solid-phase Fmoc chemistry. Cys I and Cys III were introduced as the acid labile S-trityl (Trt)-protected amino acids, which were removed from the resin by cleavage; closure of the free cysteine residues was accomplished with K_3_[Fe(CN)_6_]. Because free sulfhydryls of Cys II and Cys IV of LvIF were protected with S-acetamidomethyl (Acm) when the linear peptide was synthesized a disulfide bond was formed between Cys II and Cys IV after Acm deprotection by the second-step oxidation with I_2_ buffer. The “globular” conformation of LvIF was obtained by two-step oxidation method (Figure 2A). The fully folded parent peptides were quantitatively and qualitatively analyzed by RP-HPLC and the molecular mass of LvIF was determined by ESI-MS (Figure 2B). The molecular weight of LvIF measured by ESI-MS was 1589.8 Da, which consistent with the calculated molecular weight (1589.7 Da) (Figure 2C).

### 2.3. Effect of α-CTx LvIF on ACh-Evoked nAChR-Mediated Current

**Figure 3:**
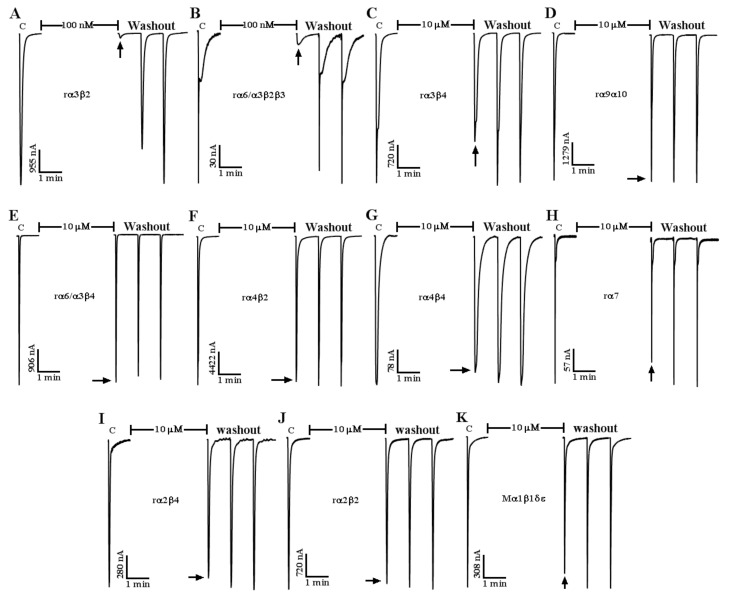
The LvIF selectively blocks rα3β2, rα6/α3β2β3, and other nAChR subtypes. *Xenopus* oocytes expressing various nAChR subtypes were exposed to 10 μM α-CTx LvIF for 5 min after control responses to ACh. The toxin was then washed out and the response to ACh was measured (arrow). The α-CTx LvIF almost completely blocked rα3β2 (**A**) and rα6/α3β2β3 (**B**), but has no obvious inhibitory effect on other nAChR subtypes, such as rα3β4 (**C**), rα9α10 (**D**), rα6/α3β4 (**E**), rα4β2 (**F**), rα4β4 (**G**), rα7 (**H**), rα2β4 (**I**), rα2β2 (**J**), Mα1β1δε (**K**), (*n* = 3).

The α-CTx LvIF is an α 4/7-CTx discovered by gene cloning in our laboratory. A two-electrode voltage clamping technique was utilized to assess the functional effects of LvIF upon a suite of nAChRs heterologously expressed in *Xenopus* oocytes. Generally, the inhibitory effect of 10 μM CTx on nAChRs is less than 50%, which was considered to have no significant effect. The results showed that the inhitibion of 10 μM α-CTx LvIF to rα3β2 and rα6/α3β2β3 nAChRs was 98.3 ± 0.6% and 98.3 ± 0.3%, respectively. The inhitibion of 10 μM LvIF to rα3β4, rα9α10, rα6/α3β4, rα4β2, rα4β4, rα7, rα2β4, rα2β2 and Mα1β1δε was 27% ± 1.21, 1.31 ± 5.2%, 2.3 ± 3.3%, 4.6 ± 2.5%, 8.6 ± 4.2%, 12.3 ± 4.2%, 9.5 ± 2.4%, 6.7 ± 4.5%, 8.7 ± 3.8%, respectively. The inhibition of LvIF on other nAChR subtypes was less than 50%. It was confirmed that α-CTx LvIF specifically targeted rα3β2 and rα6/α3β2β3 nAChR subtypes (Figure 3).

**Figure 4:**
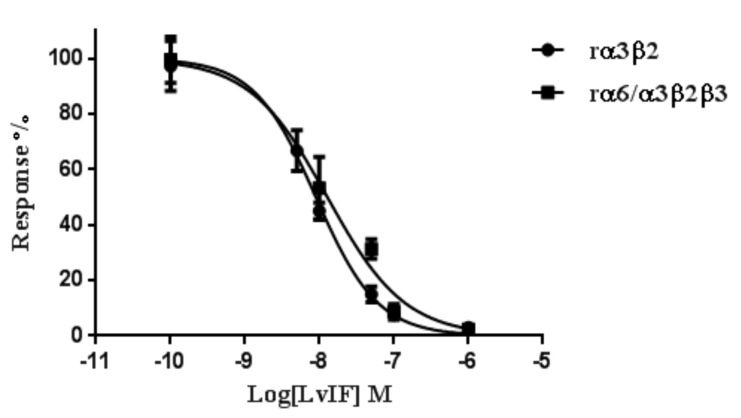
Concentration-response curve of α-CTx LvIF on rα3β2 and rα6/α3β2β3 nAChRs by α-CTx LvIF. Concentration−response of rα3β2 and rα6/α3β2β3 nAChRs exposed to LvIF and analogues. Error bars Mean ± SEM. All data were obtained 3 oocytes for each experimental determination.

To further detect the blocking effect of α-CTx LvIF on rα3β2 and rα6/α3β2β3 nAChR subtypes, the concentration-response curves of α-CTx LvIF against rα3β2 and rα6/α3β2β3 nAChRs were subsequently assessed α-CTx LvIF showed a similar dose-response curve profile in rα3β2 and rα6/α3β2β3 subtypes (Figure 4). The IC_50_ values of α-CTx LvIF for rα3β2 and rα6/α3β2β3 nAChRs was 8.98 nM and 14.43 nM, respectively (Table 1).

### 2.4. α-CTxs LvIF and MII Compete for Binding to rα3β2 nAChR

**Figure 5:**
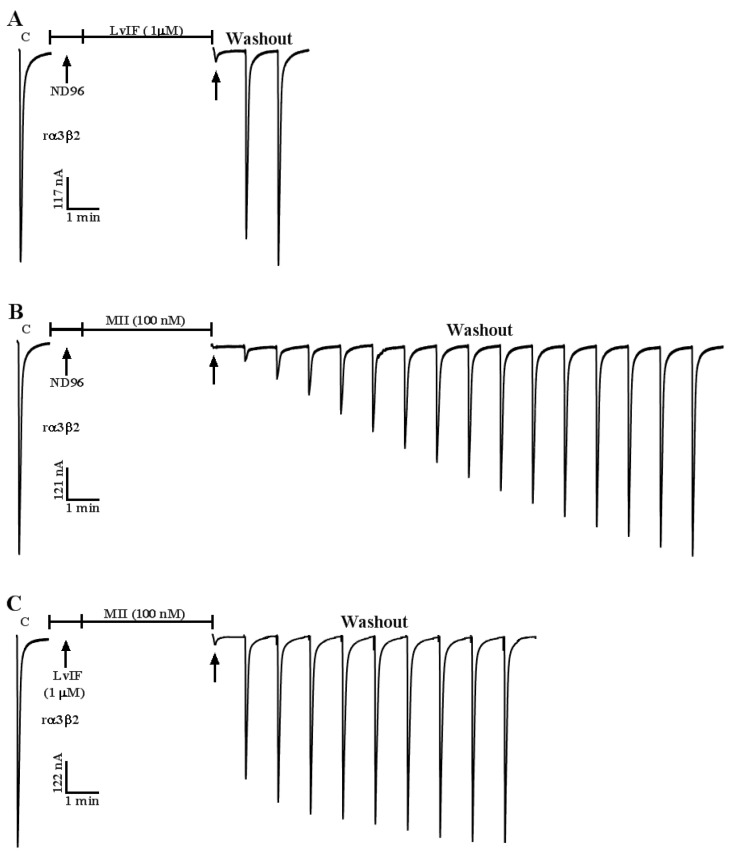
Pre-block of rα3β2 nAChR with α-CTX LvIF partially prevented the very slowly reversible block associated with α-CTx MII. In each instance, a toxin solution was applied to rα3β2 nAChRs expressed in *Xenopus oocytes*, then the toxin was washed out. The response to 1-s ACh pulse was measured as described in Materials and Methods. (**A**) 1 µM α-CTx LvIF incubated for 4 min after 1-min incubation of ND96. The ACh-evoked current traces returned to baseline level after washout for 2 min. (**B**) 100 nM α-CTx MII incubated for 4 min after 1-min incubation of ND96. The current traces returned to baseline level after washout for 15 min. (**C**) 1 µM α-CTx LvIF incubated for 1 min, followed by incubation with 100 nM α-CTx MII for 4 min. The following ACh-evoked current trace returned to baseline level after washout for 9 min. Note that pre-blockade of rα3β2 nAChR with α-CTx LvIF partially prevented the very slowly reversible block associated with α-CTx MII.

Voltage-dependent blockade suggests that ligand binds to the allosteric site or channel pore of the receptor, rather than to the ACh binding site [13]. The α-CTx LvIF blocked rα3β2 nAChR, and the current trace recovered rapidly to the baseline level after washout for 2 min (Figure 5A), while the current trace of α-CTx MII blocking rα3β2 nAChR was recovered very slowly, it took 15 min washout to restore the baseline level (Figure 5B). The binding characteristic of α-CTx was used to explore whether they have the overlapping domain with rα3β2 nAChR by competitive binding experiment. The results showed that after 1 min of the α-CTx LvIF incubation and 4 min of α-CTx MII incubation. The current trajectory could not recover to baseline level until 9 min after washout (Figure 5C). Therefore, a competitive binding relationship was confirmed between α-CTxs LvIF and MII, which bind to the partial overlapping domain in rα3β2 nAChR.

## 3. Discussion

nAChRs play a vital role in the inner synaptic signal transmission of the nervous system. The distribution of nAChRs in neurons determines their physiological and pharmacological functions. The α3 nAChR subunits mainly distribute among the central nervous system and can combine with β2 and β4 subunits to form α3β2 and α3β4 heteropentamers nAChR subtypes, respectively, which are related to a series of autonomic nerve functions with different neuropathological and physiological properties [14]. The α3β2 nAChR is one of the important subtypes in the retina, optic nerve, and striatum [15]. It has been found the α3β2 nAChR subtype is associated with analgesia [16]. The α3β4 nAChR is an important subtype which widely distributed in autonomic neurons, adrenal medulla, medial habenular nucleus, interpeduncular nucleus, pineal gland and retina. α3β4 nAChR is mainly related to physiological and pathological processes such as nicotine addiction, drug abuse and lung cancer [17,18,19,20,21]. Therefore, it is of great significance to find more pharmacological tool drugs targeting the α3β2 nAChR subtype.

The α-CTxs are a rich sources of antagonists that target α3β2 nAChR. Based on the conservation of the α-CTx gene sequence, a pair of specific primers for α-CTx were designed according to the intron sequence of α-CTx precursor gene and its 3’untranslated region (3’-UTR) sequence. A new precursor gene of α-CTx LvIF was obtained from *C. lividus* from Hainan Province by gene cloning, and its mature peptide α-CTx LvIF was further obtained. Two pairs of disulfide bonds with Cys I-Cys III and Cys II-Cys IV were formed by a two-step oxidation procedure. The blocking effect of α-CTx LvIF was detected by a two-electrode voltage clamp.

We found that α-CTx LvIF had the most potent block on rα3β2 nAChRs. It also blocks rα6/α3β2β3 but not on the other nAChR subtypes. According to previous reports, α-CTx LvIA obtained from *C. lividus* also has a blocking effect on rα3β2 and rα6/α3β2β3 nAChRs, while LvIA maintains its blocking activity against rα3β4 and rα6/α3β4 nAChRs. A serious effect of α-CTxs that target rα3β2 nAChRs has been found from other *Conus* species, such as α-CTxs MII, RegIIA, BuIA, PeIA, and GIC (Table 2) [11,22,23,24,25,26,27,28,29,30]. These α-CTxs potently inhibit the rα3β2 nAChR subtype but possess different selectivity profiles on other subtypes. They all have a conservative Ser-X-Pro-X (where X designates any different amino acid) motif in the Loop 1 sequence, which is important for α-4/7 α-CTx binding with nAChRs [31]. However, the amino acids located in Loop 2 are very important to determine their receptor subtype selectivity.

Molecular dynamics simulations of α-CTxs RegIIA and [N11A, N12A]RegIIA showed toxin binding pocket of rα3β2 nAChR, which involved in the interaction rα3 principal subunit and rβ2 complementary subunit. Some key amino acid residues in rα3 (Tyr92, Ser149, Tyr189, Cys192, and Tyr196) and β2 (Trp57, Arg81, and Phe119) subunits were identified, which would significantly affect the selectivity of RegIIA in rα3β2 nAChR [31,32,33]. The Asn 11 and Asn 12 of LvIF are the same sites as α-CTx RegIIA, while other positions are different from α-CTx RegIIA. The side chains of Ala 9 and Gly 10 of LvIF are longer than those amino acids in α-CTx RegIIA Asn 9 and Val 10, respectively. The Gln 13 of LvIF is a polar amino acid, the Asp14 is an acidic amino acid, the Pro13 of RegIIA is a non-polar amino acid, and the His14 is a basic amino acid. As a result, LvIF demonstrated higher selectivity than α-CTx RegIIA.

The α-CTx LvIA also inhibits rα3β4 nAChR subtype. It was found that the replacement of Asn 9 of α-CTx LvIA by Ala reduced the blocking activity of rα3β4 nAChR. The Arg113 of rβ4 subunit still interacted with the Val 10 and Asp 11 of α-CTx [N9A]LvIA through hydrogen bonds, while Gly 10 and Asn11 in LvIF broke the hydrogen bond with the Arg 113 of rβ4 subunit, resulting in the loss of blocking effect on rα3β4 nAChR [33,34]. Here, it is worth noting that both α-CTxs GIC and LvIF are composed of 16 amino acids and share a highly similar sequence. GIC and LvIF were identified from two different species, *C. geographus* and *C. lividus* respectively. The Asp14 of LvIF is a negative charged acidic amino acid, while the His14 of GIC is a positive charged basic amino acid at neutral pH.

**Figure 6:**
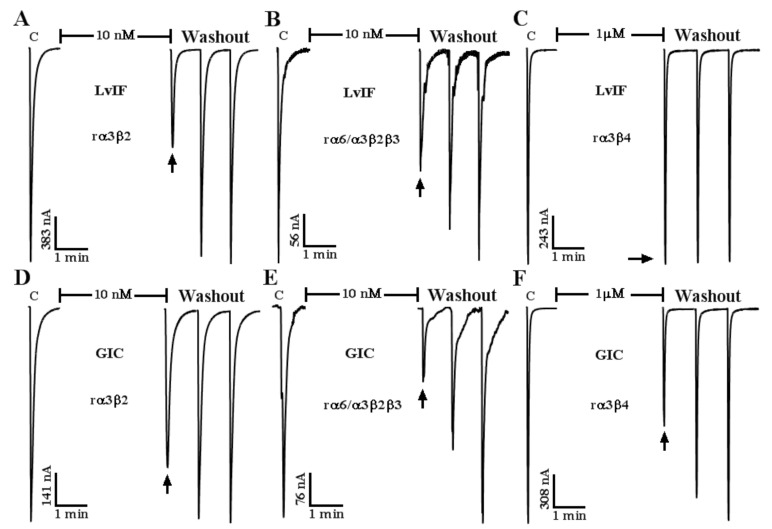
Representative current responses of α-CTx LvIF (**A**–**C**) and GIC (**D**–**F**) on rα3β2, rα6/α3β2β3, and rα3β4 nAChR subtypes respectively. LvIF and GIC showed different responses on these nAChR subtypes with different selectivity profiles, (*n* = 3).

In this work, we found that the most effective receptor for α-CTx LvIF against is rα3β2 nAChR subtype. The inhibition of 10 nM LvIF at rα3β2 and rα6/α3β2β3 nAChR subtypes were ~57% (>50%)and ~39% (<50%), respectively. The most effective receptor for α-CTx GIC against is rα6/α3β2β3 nAChR subtype. The inhibition of 10 nM α-CTx GIC on rα6/α3β2β3 and rα3β2 nAChR subtypes were ~60% (>50%) and ~43% (<50%), respectively. It is obvious that there is no inhibition of 1 μM α-CTx LvIF at rα3β4 nAChR subtype. But the inhibition of 1 μM α-CTx GIC at rα3β4 nAChR was ~50% (Figure 6). Comparison of LvIF and GIC activities on nAChRs are shown in Table 2, which are different from each other. GIC showed activity to block human and rat α4β2 and α3β4 nAChRs [28]. However, LvIF did not block α4β2 and α3β4 nAChRs (Figure 3, Table 1). GIC has similar potency on human α6/α3β2β3 and α3β2 nAChRs [30,34]. GIC showed a slightly higher potency on rat α6/α3β2β3 vs. α3β2 nAChRs (Figure 6). LvIF displayed more potent on rat α3β2 than α6/α3β2β3 nAChRs (Figure 3 and Figure 6, Table 1 and Table 2). Both the His14 of GIC and the Asp14 of LvIF are key residues to maintain their selectivity profiles on nAChR subtypes [35].

It was found that two α-CTx GIC and MII antagonize α3β2 nAChR subtype containing a rather large hydrophobic surface within the N-terminal loop region. Hydrophobic interaction is known as one of the important binding mechanisms for ligand–nAChR binding [36,37]. On the other side, two sequential asparagines Asn11/Asn12 (N/N) of Loop 2 were found in LvIF and GIC, while Glu11/His12 residues exist in MII, or, which is clustered in the middle. This finding suggests presence of such a hydrophobic surface on one side of the toxin in combination with two contiguous hydrophilic residues of loop 2 is necessary for an α-CTx to interact with the α3β2 subtype. When the amino acid in loop2 is changed, it may affect the binding to the α3β2 nAChR subtype [38]. GIC docking models with rα3β2 and rα3β4 nAChRs show that Gln13 of GIC is surrounded by Glu61, Val111, Ser113, Ser117, and Phe119 of rβ2 subunit. The corresponding residues in the rβ4 subunit are Glu62, Ile113, Arg115, Ser119, and Leu121. These residues are identical or similar to those found in rβ2 except for Arg115.

GIC still had a certain degree of blocking effect on rat α3β4 nAChR in this study (Figure 6), as well as human α4β2 and α3β4 nAChRs [28]. While LvIF did not block rα3β4 and α4β2 nAChRs at all (Figure 3 and Figure 6; Table 1). The α-CTxs GIC and LvIF only differ from the 14th-position amino acid in the peptide sequence. Therefore, we speculate that Asp14 of LvIF is a key residue to bind its receptors and decide its selection profile on nAChR subtypes. The site-directed single or multiple substitutions of amino acids can effectively improve the potency and selectivity of α-CTxs to make more effective pharmacological tools or molecular probes [39,40].

Generally, a one amino acid difference may result in changes of activity and the selection profile for an α-CTx. For example, α-CTx TxIB is a potent and high selective antagonist of α6/α3β2β3 nAChR and has no effect on other subtypes, which was found in our group [41]. When we replaced the 11th-position Lys of TxIB with Ala to form a point mutant [K11A]TxIB, its selectivity changed significantly. [K11A]TxIB displayed an obvious block on rα7 nAChR, while the wild-type TxIB has no inhibition on rα7 nAChR [42]. Similar situation occurred for α-CTx TxID vs. [S9K]TxID and α-CTx LvIA vs. [N9A]LvIA [11,35]. Therefore, a key single amino acid substitution could affect the activity and selectivity of an α-CTx, which occurred similarly for GIC and LvIF in this work.

Compared with other toxins, α-CTx LvIF has better selectivity than the others shown in Table 2. Current traces of α-CTx LvIF binding rα3β2 nAChRs were recovered to the baseline upon washout more rapidly than MII (Figure 5). This characteristic could be used for exploring whether there was an overlapping domain between LvIF and MII. Pre-block of rα3β2 nAChR with α-CTx LvIF partially prevented the slowly reversible block associated with α-CTx MII (Figure 5). So LvIF and MII may bind different sites of α3β2 nAChR but partially overlap each other. Both α-CTxs MII and LvIF belong toα4/7-CTxs and they have a Ser-X-Pro-X motif, which plays an important role in binding to rα3β2 nAChR. In addition, the first position in the sequence of the mature peptide of α-CTxs MII and LvIF are Gly. Therefore, it is speculated that the Gly-Ser-X-Pro-X motif causes the partial overlapping domain of α-CTxs MII and LvIF with rα3β2 nAChR [28,43].

According to previous studies on GIC, the amino acids that play a vital role in its activity should be His5, Ala7, and Gln13, not His14 [44]. Some other research results on α4/7-CTx also showed that the key amino acid positions are mainly focused on the Ala9, Gly10, and Asn11 amino acids [11,45]. Therefore, it is interesting that we not only found a new α-CTx highly similar to GIC in *C. lividus* but also identified that the 14th-position amino acid is also a key site that determines the activity and selectivity of the α-CTx, although LvIF is highly similar to GIC.

In summary, we identified a new α 4/7-CTx LvIF, as a selective antagonist of rα3β2 nAChRs. Characterization of LvIF in this work not only provides a new pharmacological tool but also provides new site information for ligand activity. It is helpful to design more molecular probes based on LvIF. The molecular mechanism of LvIF interaction with nAChRs and modifications will be the direction of our in-depth research in the future.

## 4. Materials and Methods

### 4.1. Materials

*C. Lividus* specimens were collected from nearby Hainan Province. Clones of rat (r) α2, α3, α4, α7 and β2, β3, β4, as well as mouse (m) α1, β1, δ, ε cDNAs were generously provided by S. Heinemann (Salk Institute, La Jolla, CA, USA). It is worth noting that that the rα6 subunit is difficult to express in vitro, so we constructed rα6/α3 chimeric subunit instead, which consisted of the N-terminal extracellular ligand-binding domain of the rα6 subunit and the remainder as the rα3 subunit segment [46]. Rα6/α3 chimera clone was generously provided by J. E. Garrett (Cognetix, Inc., Salt Lake City, UT, USA). Clones of rα9 and rα10 were kindly provided by A.B. Elgoyen (Instituto de Investigaciones en Ingeniería Genética y Biología Molecular, Buenos Aires, Argentina). C. W. Luetje (University of Miami, Miami, FL, USA) provided clones of rβ2 and rβ3 subunits in the high-expressing pGEMHE vector. The capped RNA was synthesized using the mMESSAGE mMACHINE in vitro Transcription Kit and an RNA MEGA Clear Kit, which were purchased from Thermo Fisher Scientific (Austin, TX, USA). Acetylcholine chloride, atropine, and bovine serum albumin were obtained from Sigma (St. Louis, MO, USA). Acetonitrile (ACN, HPLC grade) was purchased from Thermo Fisher Scientific (Pittsburgh, PA, USA). Trifluoroacetic acid (TFA) was purchased from Tedia Company (Fairfield, OH, USA). Vitamin C (VC), (K_3_[Fe(CN)_6_]), I_2_ and other reagents were purchased from Guangzhou Chemical Reagent Company (Guangzhou, China). Reagents for peptide synthesis were obtained from GL Biochem (Shanghai, China). All other chemicals were analytical grade and were obtained from Sigma. Reverse-phase HPLC analytical Vydac C18 columns (5 μm, 4.6 × 250 mm, 300-Å pore size; cat. no.218TP54) and preparative C18 Vydac columns (10 μm, 22 × 250 mm) were obtained from Grace Vydac (Hesperia, CA, USA). The absorbance monitor for analytical HPLC was a Waters 2996 photodiode array detector (Waters Corp., Milford, MA, USA). The female *Xenopus laevis* used for experiments were obtained from Nasco (Fort Atkinson, WI, USA) and were housed at 17 °C in our laboratory animal room and fed twice a week.

### 4.2. Identification and Sequencing of a Genomic DNA Clone Encoding α-CTx LvIF

Genomic DNA was extracted from the venom duct and gland bulb of a *C. lividus* specimen using a marine animal genomic DNA isolation Kit (Tiangen Biochemistry Ltd., Beijing, China). The procedure was described by the manufacturer in the user manual guide. The genomic DNA was used as a template for PCR amplification based on the 3′-end of the intron preceding the toxin region of α-CTx prepropeptides and the 3′-UTR (untranslated region) sequence of α-CTx prepropeptides. The sequence of the forward primer was 5′-GTGGTTCTGGGTCCAGCA-3′. The sequence of the reverse primer was 5′-GTCGTGGTTCAGAGGGTC-3′. Final PCR amplification was performed with a cycling protocol composed of an initial denaturation and preheating at 94 °C for 7 min, 35 cycles at 94 °C for 30 s, 50 °C for 1 min, 72 °C for 2 min and terminated with a final extension at 72 °C for 10 min. Positive colonies were picked and the sequence accuracy was finally determined by sequencing in Sangon Ltd. (Shanghai, China). The sequencing results were analyzed using the online tool ProP 1.0 Server to obtain conotoxin gene and precursor peptide sequences.

### 4.3. Peptide Synthesis

The α 4/7-CTxs LvIF was oxidized and folded for forming the “globular” disulfide connectivity. The procedure was described previously [47], In brief, a two-step oxidation protocol was adopted to selectively fold the peptides, which form the structure connection of Cys I-Cys III and Cys II-Cys IV. The specific steps were as follows: The 0.3 g K_3_[Fe (CN)_6_] and 0.6 g Tris were dissolved in 100 mL ddH_2_O, the 10 mg linear peptide was added after magnetic stirring, and the first disulfide bond was formed between Cys I and Cys III by stirring 45 min, at room temperature. The monocyclic peptide was purified by reversed-phase high-performance liquid chromatography (RP-HPLC) on a Vydac C18 column using a linear gradient of 10−40% buffer B (0.05% TFA, 90% acetonitrile in doubly distilled H_2_O) for 40 min and the absorbance was monitored at 214 nm. The 0.35 g I_2_ is dissolved in 8 mL ACN and the vortex vibrates until completely dissolved, then 24 mL ddH_2_O and 0.98 mL TFA are added and magnetically stirred, the purified monocyclic peptide is added drop by drop in nitrogen protection, the monocyclic peptide is catalyzed to form a second disulfide bond between Cys II and Cys IV in this solution by magnetic stirring for 15 min, and the protective group is removed, saturated VC is added drop by drop to make the solution colorless. The RP-HPLC purification steps were repeated. The bicyclic peptide with purity >95% was collected and packaged, and the trace sample solution was identified by electrospray ionization mass spectrometry (ESI-MS) mass spectrometry and analytical RP-HPLC purity identification, and then freeze-dried to the powder state by vacuum freeze-dryer.

### 4.4. cRNA Preparation and Injection

The glycerol bacteria containing a variety of nAChRs subunit gene plasmids were inoculated on solid LB medium, a colony of bacteria from a fresh solid LB plate was inoculated into LB liquid medium (containing 1% ampicillin) for overnight growth at 37 °C with shaking at 250 rpm. The bacterial solution was centrifuged at 5000× *g* to obtain the cell precipitation, and the plasmid extraction kit was used to extract the plasmid. The plasmids containing various nAChRs subunits were digested with corresponding restriction endonucleases [48,49,50,51,52,53], and then the linearized DNA products were purified by Minibest DNA fragment recovery kit (TaKaRa, Beijing, China) and used as a template for in vitro transcription. The linearized DNA templates were transcribed by using mMESSAGE mMACHINE Kit (Thermo Fisher Scientific) at 37 °C for 4 h, and the 1 µL DNase was added and treated for 30 min to hydrolyze the DNA transcription template after the completion of transcription. The cRNA product was purified by RNA MEGA Clear Kit (Thermo Fisher Scientific). The cRNA encoding various nAChR subunits were injected with equal ratios (1:1), and high-quality oocytes were selected for injection to ensure that each oocyte was injected with no less than 15 ng of cRNA. RNase contamination should be avoided during the injection. The injected oocytes were transferred to a small petri dish, and ND96 buffer (96.0 mM NaCl, 2.0 mM KCl, 1.8 mM CaCl_2_, 1.0 mM MgCl_2_, 5 mM HEPES, pH 7.1–7.5) supplemented with 10 μg/mL of penicillin, 10 μg/mL of streptomycin and 100 μg/mL of gentamicin for 2–5 days after injection.

### 4.5. Electrophysiology

Oocytes were transferred to a 50 μL cylindrical oocyte recording chamber, the holding potential was -70 mV, the internal solution of the electrode was 3 mol/L KCl, resistance was about 0.5–2 MΩ, and the perfusion solution was ND96 buffer and perfused at a rate of ~2 mL/min with ND96 buffer (supplemented with 1 μM atropine and 0.1 mg/mL of bovine serum albumin by a gravity fed perfusion system. The mother liquid of α-CTx LvIF was diluted to a series of concentration gradients with ND96 buffer, and the mother solution of acetylcholine (Ach) was diluted to the appropriate concentration with ND96 buffer, and the acetylcholine-evoked (ACh-evoked) currents were recorded with a two-electrode voltage-clamp amplifier Axon 900A (Molecular Devices Corp., Sunnyvale, CA, USA). In the blank control experiment, ACh-evoked currents were recorded in response to ND96. The potency of α-CTx LvIF was determined by comparing the ACh-evoked currents after 5 min incubation with different concentrations of conotoxin solution. The control currents evoked by ACh were repeated at least three times to obtain the average peak current. Each concentration LvIF was tested on three to six oocytes. There is a 1-s pulse of 10 μM ACh in one sweep corresponding to the Mα1β1δε and rα9α10 subtypes, 200 μM ACh is for rα7 subtype, and 100 μM for all the other subtypes, respectively [54]. Peak current amplitude was measured and analyzed by Clampfit 10.2 software (Molecular Devices Corp., Sunnyvale, CA, USA) before and after incubation of the peptide. All recordings were conducted at room temperature (20–25 °C).

### 4.6. Data Analysis

For the baseline response, at least three ACh responses were averaged. The average of α-CTx LvIF on ACh-evoked steady-state currents was defined as peak current amplitudes, and the value was divided by the pre-toxin baseline value to yield a “% response”. Each data point of a dose-response curve represents the Mean ± SEM of at least three oocytes. The dose-response data were fit to the equation: response (%) = 100/[1 + ([toxin]/IC_50_)^Hill slope^], where Hillslope is the Hill coefficient and IC_50_ value is the antagonist concentration giving a half-maximal response, by nonlinear regression analysis using GraphPad Prism 6.0 (GraphPad Software, San Diego, CA, USA).

## Figures and Tables

**Table 1:** The IC_50_ and Hill slope values of α-CTx LvIF inhibit different nAChR subtypes.

Subtype	IC_50_ (nM) ^a^	Hill Slope ^a^
rα3β2	8.98 (7.73–10.43)	1.0 (0.9–1.2)
rα6/α3β2β3	14.43 (10.24–20.39)	1.0 (0.7–1.1)
mα1β1δε	>10,000 ^b^	
rα2β2	>10,000 ^b^	
rα2β4	>10,000 ^b^	
rα3β4	>10,000 ^b^	
rα4β4	>10,000 ^b^	
rα4β2	>10,000 ^b^	
rα6/α3β4	>10,000 ^b^	
rα7	>10,000 ^b^	
rα9α10	>10,000 ^b^	

^a^ Values in parentheses are 95% confidence interval (C.I.). ^b^ Less than 50% blocking at 10 μM. All receptors are of rat (r) origin, except α1β1δε which is of mouse (m) origin.

**Table 2:** Sequence comparison of α-CTx LvIF with α-CTxs that have similar activity for α3β2 nAChR.

α-CTx	Organism	Sequence ^a^	Target ^b^	Ref.
LvIF	*C. lividus*	G**CC**SHPA**C**AGNNQDI**C**#	rα3β2 > rα6/α3β2β3	This work
RegIIA	*C. regius*	G**CC**SHPA**C**NVNNPHI**C**#	rα3β2 > rα7 > rα6/rα3β2β3 > rα3β4 > rα6/α3β4	[22]
MII	*C. magus*	G**CC**SNPV**C**HLEHSNL**C**#	rα6/α3β2β3 > rα3β2 > rα6/α3β4	[23,24]
LvIA	*C. lividus*	G**CC**SHPA**C**NVDHPEI**C**#	rα3β2 > rα6/α3β2β3 > rα6/α3β4 > rα3β4	[11,25]
BuIA	*C. bullatus*	G**CC**STPP**C**AVLY---**C**#	rα2β2 > rα2β4 > rα4β4 > rα3β2 > rα3β4 > rα6/α3β4 > rα6/α3β2β3	[26]
PeIA	*C. pergrandis*	G**CC**SHPA**C**SVNHPEL**C**#	rα9α10 > rα6/α3β2β3 > rα3β2 > rα6/α3β4 > rα3β4	[27]
GIC	*C. geographus*	G**CC**SHPA**C**AGNNQHI**C**#	rα6/α3β2β3 ≥ rα3β2 > rα4β2 > rα3β4	This work
			hα6/α3β2β3 ≈ hα3β2 > hα4β2 > hα3β4	[28,29,30]

^a^ The Cys residues that participate in the Framework I scaffold are in boxed. Conserved positions are on a light gray background. # Represents C-terminal carboxamide ^b^ r represents Rat and h represents human.

## Data Availability

The data presented in this study are available on request from the corresponding author. The data are not publicly available due to patent protection.
